# Intrauterine Growth Restriction and Placenta Previa: A Clinical Case Report of an Advanced-Age Pregnancy

**DOI:** 10.7759/cureus.54896

**Published:** 2024-02-25

**Authors:** Srihita Patibandla, Ali Z Ansari, Samuel F Brown

**Affiliations:** 1 Obstetrics and Gynecology, William Carey University College of Osteopathic Medicine, Hattiesburg, USA; 2 Obstetrics and Gynecology, South Central Regional Medical Center, Laurel, USA

**Keywords:** maternal-fetal medicine, cesarean section (cs), congenital abnormalities, high-risk obstetrics, low pre pregnancy bmi, maternal age, cigarette smoking, single umbilical artery, placenta previa, intrauterine growth restriction (iugr)

## Abstract

Exploring the intricacies of managing high-risk pregnancies complicated by intrauterine growth restriction (IUGR), placenta previa, and a single umbilical artery requires a comprehensive understanding of their etiologies, mechanisms, and treatment recommendations. This case report delves into the clinical course of a 34-year-old smoker with a pre-pregnancy body mass index of 14.2 kg/m^2^, shedding light on the considerations posed by a pregnancy in which several risk factors are superimposed on one another. IUGR, affecting 10%-15% of pregnancies, elevated the risk of adverse outcomes during labor and delivery, necessitating careful antenatal monitoring. Placenta previa, with an incidence of 0.3% to 2% in pregnancies, introduced further complications impacting delivery modes and raising the risk of hemorrhage. This report aims to showcase the interconnectedness between these various obstetrical complications and risk factors, to guide maternal-fetal-medicine specialists in making informed decisions during the management of high-risk pregnancies.

## Introduction

Within the field of maternal-fetal medicine, the management of pregnancies complicated by intrauterine growth restriction (IUGR), a two-vessel cord, and placenta previa demands a thorough understanding of their pathophysiology and associated risk factors. This case report explores the clinical course of a 34-year-old female with low pre-pregnancy body mass index (BMI), highlighting the complexities of a pregnancy marked by both IUGR and placenta previa. IUGR is a complex but common problem encountered in obstetrics, with an incidence of 10%-15% [1,2]. It is characterized as the pathological inhibition of fetal growth within the uterus, leading to the failure of the fetus to reach full growth potential [2]. IUGR increases the risk of adverse outcomes during labor and delivery, predisposing infants to complications such as intrapartum asphyxia, preterm delivery, and associated risks such as respiratory distress syndrome, intraventricular hemorrhage, and necrotizing enterocolitis [1]. Infants will often have low Apgar scores and umbilical cord pH below 7.0, necessitating intubation, and experience complications such as seizures, sepsis, and death [1]. Risk factors for IUGR include maternal factors such as extremes of age, pre-eclampsia, low pre-pregnancy weight, and infertility; fetal factors such as genetic causes, congenital malformations, and infection; and placental factors such as placental insufficiency or abnormal implantation [1,3]. 

Placenta previa, another critical aspect of this case, involves the abnormal implantation of the placenta, resulting in partial or complete covering of the internal cervical os [4]. This is a condition that affects 0.3% to 2% of pregnancies in the third trimester and is associated with endometrial damage and uterine scarring [4]. Risk factors include advanced maternal age, multiparity, previous cesarean section (C-section), abortion, and smoking and cocaine use during pregnancy. Additionally, there is an association with male fetuses [5]. The presence of placenta previa not only raises concerns regarding the mode of delivery but also introduces potential complications to mother and fetus such as maternal hypertension, preeclampsia, IUGR, placental abruption, preterm delivery, antepartum hemorrhage, and perinatal mortality [6,7]. These risks are further complicated by fetal complications such as the IUGR and a two-vessel cord in this case. Understanding the risk factors associated with these conditions is imperative in providing adequate antenatal care. Through a discussion of this patient’s presentation, this report aims to highlight the interplay between the various obstetrical complications surrounding the patient’s pregnancy. Having a comprehensive understanding of the pathophysiology of these conditions and their management guidelines is necessary for optimizing maternal and fetal outcomes.

## Case presentation

The patient was a 34-year-old gravida 2, para 1, white female with a past medical history significant for a BMI of 14.2 kg/m^2^. The patient had an uncomplicated pregnancy six years ago, followed by a full-term vaginal delivery. She desired infertility after her current pregnancy. Her last menstrual period was on June 1 of the previous year. She had a normal Papanicolaou (PAP) smear three years ago. She did not have any personal history or family history of neural tube defects, congenital heart defects, Down syndrome, sickle cell disease or trait, cystic fibrosis, metabolic disorders, genital herpes, prior group B strep (GBS) positive pregnancy, HIV, and mental retardation or autism. She did have an extensive smoking history and continued to smoke over ¼ packs per day throughout her current pregnancy. She was also sexually active during the initial 21 weeks of her pregnancy.

Her fetal anatomy ultrasound (US) performed at 21 weeks gestation revealed a fetus in a breech position with posterior placenta previa, and the patient was advised to begin strict pelvic rest and avoid intercourse. At 27 weeks gestation, the patient’s growth scan revealed a small lag in the fetal growth, and the fetus was in vertex presentation. At 31 weeks gestation, the fetus was noted to have a two-vessel cord, estimated fetal weight (EFW) at 19.6th percentile, head circumference (HC) below 1st percentile, femur length (FL) at 1st percentile, biparietal diameter (BPD) below 1st percentile, and continued placenta previa. The length of the cervix was 5.2 cm. The fetal heart rate was at 136 bpm. The patient was instructed to notify the clinic and go to the emergency department if any bleeding was noted and was scheduled for a primary C-section at 36-37 weeks gestation. Another sonogram performed at 35 weeks and 2 days gestation revealed fetal growth at the 4th percentile. However, the fetus’s biophysical profile (BPP) was reassuring with a score of 8/8. The patient was given her first 12 mg intramuscular dose of betamethasone acetate and sodium phosphate steroids for promoting fetal lung maturity. She received her second dose of steroids the following day. Another sonogram revealed continued IUGR at the 4th percentile, with normal fetal assessments and normal amniotic fluid levels. The risk of intrauterine fetal demise was determined to outweigh the risks associated with prematurity, and the patient was scheduled to proceed with her C-section.

The patient arrived at the hospital at 35 weeks and 5 days gestation with a BMI of 17.6 kg/m^2^ for a primary C-section, secondary to placenta previa and IUGR. The physical examination of the patient was unremarkable, and the pelvic examination was deferred due to the placenta previa. No bleeding was noted. Nonstress testing (NST) of the fetus was reactive and showed a category 1 tracing with a heart rate of 135 bpm, moderate variability, and no decelerations.

The C-section proceeded without complications. The operation was performed through a low-transverse uterine incision. The amniotic bag was artificially ruptured, and clear amniotic fluid was noted. The baby had a nuchal cord, which was manually reduced with no difficulty. Cord blood gases revealed a cord pH of 7.2, within the normal range. After delivery of the infant, the placenta previa was manually extracted and sent off for pathological evaluation. The placenta was grossly normal-appearing with a two-vessel cord. After the closure of the uterus, O’Leary stitches were placed around the uterine vessels bilaterally with 1-0 chromic sutures due to the possibility of postpartum bleeding. Grossly normal-appearing fallopian tubes, uterus, and ovaries bilaterally were sent for pathological evaluation for potential malignancies or abnormalities. The remainder of the operation proceeded uneventfully. The viable male infant weighed 4 pounds 11 ounces, with Apgar scores of 9/9. The placental pathology report showed mild meconium staining of the membranes, mild inflammation of the decidualized layer of the membranes beneath the chorion, sub-fetal plate fibrin plaque formation, and irregular cotyledons consistent with the patient’s clinical history of placenta previa. Following delivery, the patient was followed by a nutritionist due to her BMI <18.5 kg/m^2^ and had an uncomplicated postoperative recovery period. Labs throughout the patient’s clinical course are listed in** **Table 1.

**Table 1:** Overview of relevant labs, physical exam findings, and studies performed throughout the patient's clinical course.

Date	2/6, 6:30 am	2/6, 7:44 am	2/6, 9:13 am	2/7, 9:10 am	2/8, 8:30 am
Stage of pregnancy	Prepartum	Prepartum	Recovery	Postpartum	Postpartum
White blood cells/mcL	15,000	-	-	-	-
Hemoglobin (g/dL)	12.6	-	-	9.3	-
Hematocrit (%)	37.5	-	-	27.9	-
Platelet count/mcL	223	-	-	-	-
Rapid plasma reagin test	Nonreactive	-	-	-	-
Blood type/Rh	O positive	-	-	-	-
Antibody screen	Negative	-	-	-	-
Uterine activity					
Monitor mode	External US	External US	-	-	-
Frequency (minutes)	Irregular	2-3	-	-	-
Duration (seconds)	Irregular	60-120	-	-	-
Pain					
Pain scale	0/10	0/10	3/10	3/10	4/10
Pain type	None	None	Cramping	Cramping, ache	Ache
Pain presence	None	None	Intermittent	Intermittent	Intermittent
Pain location	None	None	Abdomen	Abdomen	Abdomen, perineum
Fetal assessments					
Heart rate (bpm)	140	135	-	-	-
Variability	Moderate 6-25 bpm	Moderate 6-25 bpm	-	-	-
Accelerations (bpm x seconds)	15 x 15	15 x 15	-	-	-
Decelerations	None	None	-	-	-
Category	Category 1	Category 1	-	-	-

## Discussion

IUGR remains a topic of extensive research surrounding its pathophysiology. Per the American College of Obstetricians and Gynecologists, IUGR is characterized by EFW below the 10th percentile [1]. IUGR has traditionally been divided into symmetrical (early) and asymmetrical (late), depending on the timing of the cause of fetal insult [1,8]. Symmetrical IUGR results in an infant with uniformly small proportions and normal HC/abdominal circumference (AC) and FL/AC ratios [8]. This may occur with an insult early in pregnancy such as protein deficiency or placental insufficiency, causing a proportional decrease in cell number and size, affecting both head and body size [1,8]. In contrast, asymmetrical IUGR is often a result of late-pregnancy insults directing oxygen and nutrients preferentially to the brain, known as *brain sparing*. The fetal brain, which is then relatively larger than the liver, is protected from the full effects of growth restriction [1,8]. The focus of prenatal care in pregnancies with IUGR is the identification of fetuses who are at high risk of complications or fetal demise and thus would benefit from early delivery. The key parameters for fetal surveillance are the EFW, Doppler ultrasonography, and fetal assessments like the BPP and NST [9]. Antenatal steroids are recommended for promoting fetal lung maturity [10]. Intrapartum administration of magnesium sulfate to mothers with IUGR infants born before 29 weeks gestation is also shown to decrease the odds of composite death or significant neurodevelopmental impairment [11]. Taking these factors into consideration is critical in the optimal management of IUGR pregnancies to prevent adverse outcomes.

Placentas are classified into three subgroups depending on their location from the cervical os; placenta previa (placenta covering the cervical os), low-lying placenta (edge of placenta ≤ 20 mm from cervical os), or normally located placenta (edge of placenta > 20 mm from cervical os) [12]. The majority of cases of low-lying placentas self-resolve by the third trimester as the placenta grows towards the increased blood supply at the fundus of the uterus, leaving the distal portion with poorer blood supply to atrophy and shrink [4]. At the same time, the low uterine segment may also grow, increasing the distance further between the lower margin of the placenta and the cervical opening [4]. Patients with a history of antepartum hemorrhage, a thick placental edge covering or near the cervical os, short cervical length (<3 cm with placenta previa or <2 cm with low-lying placenta), and a previous C-section delivery are at increased risk for requiring urgent or preterm cesarean delivery [12]. In a meta-analysis by Shobeiri and Jenabi [13], smoking during pregnancy was shown to be associated with an increased risk of placenta previa. In another meta-analysis by Balayla et al. [14], placenta previa was shown to be associated with a mild increase in the risk of the fetus developing IUGR. The primary goals of management for asymptomatic patients are to determine whether the previa resolves over time, determine if the placenta is adherent to the endometrium (placenta accreta spectrum), reduce bleeding risk, and determine optimal delivery time [15]. Alternatively, the goals of managing patients with an acute bleed are to maintain hemodynamic stability and determine if an emergency C-section is indicated [14,15]. 

Prepregnancy BMI plays a significant role in influencing various physiological processes that occur throughout pregnancy. Pregnant mothers often adjust their diet throughout pregnancy, occasionally falling short of maintaining an adequate, quality diet for gestational weight gain (GWG) [16]. Research indicates that only about 1/3rd of women in the United States achieve the recommended GWG [16]. The Institute of Medicine (IOM) guidelines for weight gain in pregnancy are outlined in Table 2 [17]. In various studies of pregnant women in the overweight or obese categories, weight loss of ≤5 kg was found to be associated with an increased risk of the infant being small of gestational age (SGA) with decreased neonatal fat mass, lean mass, and HC [18,19]. However, the negative consequences associated with high BMI and excess weight gain in pregnant overweight or obese women outweigh the risks associated with inadequate GWG [17]. In pregnant women in the underweight category with BMI < 18.5 kg/m^2^, insufficient weight gain is associated with SGA as well, in addition to premature rupture of membranes, anemia, and IUGR [20,21]. Low BMI in general, regardless of how much weight is gained during pregnancy, is also associated with preterm delivery and low birth weight [22]. However, some studies show that low BMI is associated with better pregnancy outcomes than normal BMI, such as less risk of preeclampsia and emergency C-section [23]. Excessive pregnancy weight gain in all women is suggested to be associated with an increased risk of developing diabetes and cardiovascular disease, neonatal seizures, meconium aspiration syndrome, low Apgar scores, and large for gestational-age babies [24]. Additionally, women who gained excessive weight during pregnancy had an increase of 0.72 kg/m^2^ in long-term BMI compared to women who gained weight within the IOM guidelines [25]. Obesity in particular is associated with gestational diabetes, hypertension, preeclampsia, thromboembolism, congenital fetal anomalies, macrosomia, stillbirth, shoulder dystocia, and preterm delivery [26]. Ensuring appropriate prepregnancy weight and intra-pregnancy weight gain is critical in all women for improving maternal and neonatal outcomes, with the risks overall being higher with obesity or excess weight gain compared to the alternative. 

**Table 2:** Institute of Medicine weight gain recommendations for pregnancy. ^*^Body mass index is calculated as weight in kilograms divided by height in meters squared or as weight in pounds multiplied by 703 divided by height in inches. The units listed in the table are in kg/m^2^. ˡCalculations assume a 1.1-4.4 lb weight gain in the first trimester. Adapted from the American College of Obstetricians and Gynecologists: Weight gain during pregnancy: Committee opinion no. 548. Obstet Gynecol. 2013, 121:210-2.

Prepregnancy weight category	Body mass index*	Recommended range of total weight (lb)	Recommended rates of weight gainˡ in the second and third trimesters (lb) (mean range [lb/week])
Underweight	<18.5	28-40	1 (1-1.3)
Normal weight	18.5-24.9	25-35	1 (0.8-1)
Overweight	25-29.9	15-25	0.6 (0.5-0.7)
Obese (includes all classes)	30 and greater	11-20	0.5 (0.4-0.6)

A significant finding in the patient case presented was the presence of a two-vessel cord in the neonate, which refers to a variation in umbilical anatomy in which only a single umbilical artery (SUA) is present. Research regarding the significance of the finding and its implications is ongoing, with 5.9% of fetuses diagnosed with SUA [27]. In normal development, the umbilical cord consists of one vein and two arteries. Three theories have been proposed regarding the mechanism of SUA development: (1) primary agenesis of one umbilical artery; (2) secondary atrophy or atresia of a previously normal umbilical artery; and (3) persistence of the original allantoic artery of the body stalk [28]. In a study by Hua et al. [29], SUA was found to be associated with IUGR, increased risk of renal anomalies, and cardiac anomalies. In a meta-analysis by Kim et al. [30], pregnancies with SUA had increased risk for SGA, preterm birth, pregnancy-induced hypertension, neonatal intensive care unit admission, and perinatal mortality. Ebbing et al. [31] also found a strong association of SUA with gastrointestinal atresia or stenosis, trisomy 13, and trisomy 18. Other associations include placental abruption, placenta previa, cord collapse, low Apgar scores, and polyhydramnios/oligohydramnios [27]. When the finding of an SUA is present in conjunction with other anomalies, a fetal karyotype is recommended due to the increased risk of aneuploidy [32]. The pregnancy should also be carefully monitored to promptly diagnose other associated conditions seen with abnormal amniotic fluid volume [32]. Understanding anatomical anomalies noted during pregnancy surveillance is important in allowing mothers to make informed decisions regarding the care of themselves and their newborns.

Advanced maternal age (AMA) is generally considered to be age ≥ 35 years. The patient in the case presented reached the AMA category during her pregnancy. AMA is associated with increased risk for operative and cesarean deliveries, trisomy 21 and chromosomal anomalies, stillbirth, preterm birth, placenta previa, macrosomia, large for gestational age babies, and risk of fetal death from asphyxia [33,34]. Prenatal screening for aneuploidy and a thorough second-trimester fetal anatomy ultrasound are recommended for all AMA pregnancies to screen for anatomical anomalies [35]. Prophylaxis with low-dose aspirin is also recommended in patients of AMA who have at least one other moderate risk factor for developing preeclampsia such as BMI > 30 kg/m^2^, family history of preeclampsia, or nulliparity [36]. Delivery is recommended at 39 weeks gestation in this age group due to the increased risk of fetal mortality with each additional week of expectant management [37].

The presented case demonstrates the complexity of the interconnection of risk factors present in the patient. The patient was unsuccessful in smoking cessation and smoked one-fourth pack per day throughout her pregnancy, despite extensive counseling regarding its associated risks. This, compounded with the fact that the patient had reached AMA through the duration of her pregnancy, was the possible underlying catalyst of her placenta previa [13,34]. As discussed previously, placenta previa is one of the underlying risk factors for developing IUGR [14]. This may be a result of compromised blood flow to the placenta or uterus, affecting the supply of oxygen and nutrients to the growing fetus [1,8]. In a study by Odibo et al., AMA was also shown to be an independent risk factor for IUGR [38]. These two factors likely worked in conjunction with the patient’s low prepregnancy weight and extensive smoking history to contribute to the development of fetal growth restriction [20,39]. The patient, with a BMI < 18.5 kg/m^2^ before pregnancy, only gained 18 pounds throughout her pregnancy course, which is only 64% of the minimum recommended amount per IOM guidelines. The newborn IUGR may be an indication of a more serious underlying congenital anomaly such as trisomy 21, as suggested by the intraoperative finding of a two-vessel cord [31]. The SUA is yet another factor that could have contributed to, or is associated with, the development of placenta previa in the mother [27]. The interconnectedness between all these compounding factors highlights the importance of a comprehensive understanding of their collective impact. This understanding will guide the personalized management and perinatal monitoring that is necessary throughout such high-risk pregnancies. This kind of comprehensive approach is necessary for optimizing outcomes for the mother and fetus. 

## Conclusions

The presented case highlights the challenging management of a pregnancy marked by IUGR, placenta previa, and a two-vessel cord. The patient’s history, including a low prepregnancy BMI, advanced maternal age, and persistent smoking, further added to the complexity of decision-making involved in the case. This amalgamation of multiple risk factors to both mother and fetus necessitates careful antenatal monitoring, specialized screening and interventions, and the timely decision for a primary C-section at 35 weeks and five days gestation. The successful delivery of a viable male infant shows the importance of thorough maternal-fetal care. The postoperative period, involving follow-up with a nutritionist, also highlights the need for multidisciplinary care in high-risk pregnancies. Ultimately, the goal is to ensure the health and wellness of both the patient and her baby.
